# Electrochemical Sensor Based on Co-MOF for the Detection of Dihydromyricetin in *Ampelopsis grossedentata*

**DOI:** 10.3390/molecules30010180

**Published:** 2025-01-05

**Authors:** Xiaojing Si, Yue Huang, Mei Han, Liqiang Luo

**Affiliations:** 1 Department of Food Science, Shanghai Business School, Shanghai 200235, China; 21190008@sbs.edu.cn (X.S.); 21180003@sbs.edu.cn (M.H.); 2 College of Sciences, Shanghai University, Shanghai 200444, China

**Keywords:** dihydromyricetin, determination, electrochemical sensor, metal-organic framework, *Ampelopsis grossedentata*

## Abstract

Dihydromyricetin (DMY), as the main active ingredient in *Ampelopsis grossedentata*, is a naturally occurring flavonoid that has attracted extensive attention for its multiple biological activities. For the quick and accurate measurement of DMY, a novel electrochemical sensor based on a glassy carbon electrode (GCE) modified with a cobalt metal-organic framework (Co-MOF) was proposed in this work. The Co-MOF was synthesized via a single-step hydrothermal process using Co(NO_3_)_2_·6H_2_O. Fourier infrared spectroscopy, X-ray photoelectron spectroscopy and scanning electron microscopy were used to study the morphology and structure of the synthesized Co-MOF. Utilizing differential pulse voltammetry and cyclic voltammetry methods, the effectiveness of DMY electro-oxidation on the Co-MOF/GCE was examined. The results showed that, in comparison to the bare GCE, the electro-oxidation peak current of DMY was considerably increased by the Co-MOF/GCE. The detection limit was 0.07 μM, and the peak current demonstrated two linear relationships in the ranges of 0.2−20 μM and 20−100 μM, with the linear equations of Ip (μA) = 0.4729c (μM) + 1.0822 (R^2^ = 0.9913) and Ip (μA) = 0.0939c (μM) + 8.4178 (R^2^ = 0.9971), respectively. The average DMY content in *Ampelopsis grossedentata* samples was measured to be 3.275 μM, with a good recovery of 108.27% and a relative standard deviation value of 3.46%. The proposed method is simple, rapid and sensitive and can be used for the determination of DMY in *Ampelopsis grossedentata*.

## 1. Introduction

*Ampelopsis grossedentata*, the botanical name of the serpentine grape in the genus Serpentine, family Vitis vinifera, also known as berry tea or vine tea, is mainly distributed in the southern provinces of China and mostly concentrated or scattered in mountainous bushes or woods with high humidity [1]. The leaves of *Ampelopsis grossedentata* have been widely used in China as a health tea and herbal medicine for more than 1200 years [2,3].

Dihydromyricetin (DMY), the main active ingredient in *Ampelopsis grossedentata*, is a naturally occurring flavonoid that has attracted extensive attention for its multiple biological activities [4], such as its antineoplastic [5], antidiabetic [6], anti-inflammatory [7], antioxidant [8], antibacterial [9], antineoplastic [10], neuroprotective [11] and enteroprotective [12] properties. The content of DMY is used as a standard substance for evaluating the total flavonoid content in *Ampelopsis grossedentata*, which has become one of the indicators for evaluating the quality of *Ampelopsis grossedentata*, and the content is also often shown in the labeling information of commercial *Ampelopsis grossedentata* products. DMY has a wide range of application prospects in food development and processing. For example, DMY has strong antioxidant activity, and can be added in the process of cookie making. The color and aroma of the product have a special beneficial effect, and the texture does not change significantly, but it may weaken the role of lipid and protein oxidation [13,14]. Therefore, it is an urgent problem to quickly and conveniently detect the content of DMY for determining the quality of *Ampelopsis grossedentata*.

**Figure 1:**
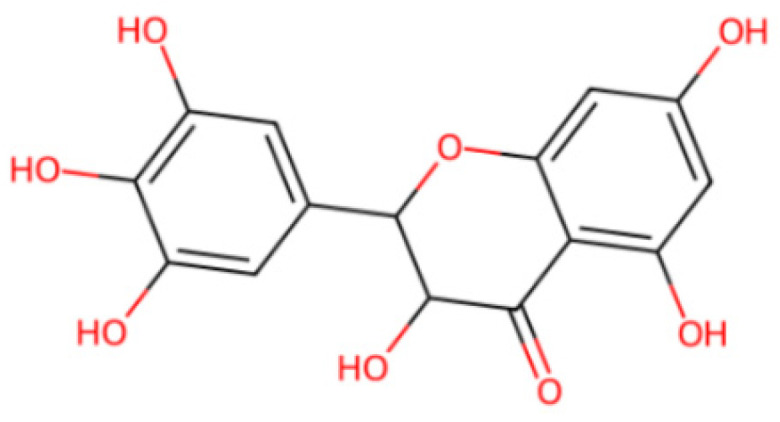
Structural formula of DMY.

In recent years, chromatography and capillary electrophoresis have been developed for the determination of DMY in plants, foods and biological samples [15,16]. However, these methods require expensive instrumentation, complicated sample pretreatment processes and time-consuming detection procedures, which are not suitable for routine large-batch determination of the DMY content in *Ampelopsis grossedentata* for product screening and grading. In this regard, the identified and quantified methods based on electrochemical sensors have progressed rapidly due to the advantages of the fast analytical response, low dose requirement, simple sample pretreatment process, high precision and sensitivity, which have been applied in the food, environment, biomedicine and biopharmaceutical areas [17,18,19]. As known, the chemical structure of DMY (as shown in Figure 1) contains several hydroxyl groups, which can generate electrochemical signals. Hence, electrochemical sensors maybe a better choice for the rapid determination of DMY in *Ampelopsis grossedentata*.

It is essential to build sensitive and effective electrochemical sensors for quick determination. A wide range of micro- and nanomaterials with various properties have been used to build electrochemical sensors. These materials include polymers, carbon nanomaterials (carbon nanotube, graphene, g-C_3_N_4_, etc.), quantum dots, metal and metal derivatives and metal-organic frameworks (MOFs) [20,21]. The DMY content in *Ampelopsis grossedentata* can be successfully determined using the previously reported DNA immobilized ethylenediamine/polyglutamic modified electrode [22], nafion/single-walled carbon nanotube modified glassy carbon electrode (GCE) [23] and graphene–nafion composite film modified electrode [24]. However, there have not yet been any reports on the determination of DMY using electrochemical sensors based on MOFs.

MOFs with redox-active metal ions and a variety of organic linkers have been widely investigated as potential prospective electrode materials in chemical sensors because of their large specific surface area, adjustable aperture size, different topological structures, strong catalytic efficiency and good stability [25,26]. Generally, MOFs are composed of a variety of metals, including Zr, Cu, Ni, Fe and Co [27,28,29]. Compared to other metal-based MOF materials, Co in various oxidation states can result in surface Faradaic reactions, good electrochemical activity and exceptional thermal stability. Usually, cobalt salt and organic ligand are the main materials for the preparation of Co-MOF. Cobalt nitrate is one of the commonly used cobalt salts, and organic ligand is an important component of the coordination reaction with cobalt ion. The choice of organic ligand directly affects the structure and properties of Co-MOF, in which 1,4-benzenedi-carboxylic acid has a stable benzene ring structure and a variety of coordination modes of carboxylic acid groups. Thus, Co-MOF has wide application potential in the fields of gas storage and separation, catalysis, drug delivery and sensors [30].

In this work, Co-MOF was synthesized through the single-step hydrothermal method. The composition, structure and morphology of the synthesized Co-MOF were characterized by X-ray photoelectron spectroscopy (XPS), X-ray diffraction (XRD), Fourier infrared spectroscopy (FTIR) and scanning electron microscopy (SEM). For application, the synthesized Co-MOF was modified on GCE to increase the selectivity and sensitivity for the detection of DMY content in *Ampelopsis grossedentata*. This work widens the application of Co-MOF in the electrochemical analysis of DMY in *Ampelopsis grossedentata*.

## 2. Results and Discussion

### 2.1. SEM, XPS, FTIR and XRD Characterization of Co-MOF

**Figure 2:**
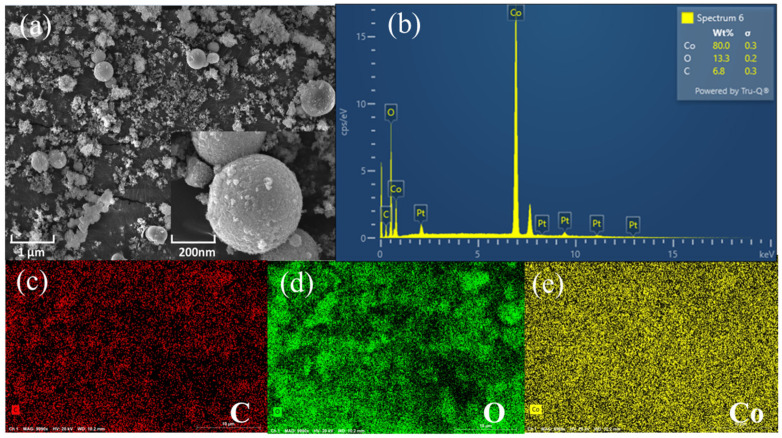
SEM image (**a**) and EDS image of Co-MOF (**b**–**e**).

The composition and morphology of the synthesized Co-MOF were analyzed by SEM. The SEM and energy dispersive spectroscopy (EDS) image of Co-MOF at different resolutions are shown in Figure 2. As indicated from Figure 2a, the acquired Co-MOF is porous and forms regular spherical crystals with an average size of 300–400 nm in diameter, illustrating the nanostructure of the material.

A part of the Co-MOF material was selected for the EDS spectroscopy test to further verify the elemental composition of the material. The distribution of C, O and Co elements can be observed from Figure 2b–e. The nanomaterial consists of three elements, C, O and Co, with the contents of 6.8%, 13.3% and 80.0%, respectively, indicating that Co metal has been doped into the organic framework material, and the Co-MOF nanocomposite material with an ordered arrangement has been successfully synthesized. This result is consistent with that of previous reports [31].

**Figure 3:**
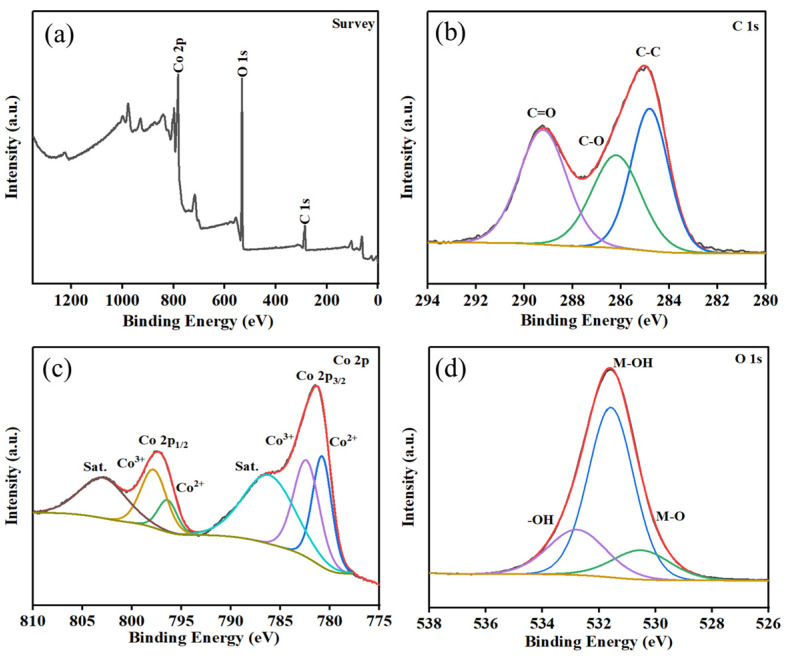
XPS of (**a**) survey spectrum, (**b**) C, (**c**) O and (**d**) Co.

XPS was used to analyze the chemical bond states of Co-MOF. Based on the Co-MOF thin film’s XPS survey spectrum (Figure 3a), we can see that there were Co 2p, O 1s and C 1s peaks with binding energies of 782, 532 and 286 eV, respectively; and almost no impurity peaks were observed, confirming that the Co-MOF contains Co, O and C elements. Furthermore, as displayed in the high-resolution XPS spectrum (Figure 3b), the spectrum of C 1s can be indexed into three peaks at 295.9, 286.9 and 284.6 eV, which correspond to the bonds of C=C, C–O and C=O, respectively [32]. In Figure 3c, the spectrum of O 1s is asymmetric, and two symmetric peaks (532.9 and 530.2 eV) can be fitted. This indicates the presence of adsorbed oxygen of the surface hydroxyl group and lattice oxygen of Co-MOF, respectively. The spectrum of Co 2p (Figure 3d) consists of two separate symmetric peaks. At binding energies of 781.8 eV (Co 2p_1/2_) and 798.0 eV (Co 2p_3/2_), a two-state spectrum of Co 2p can be observed, which is close to the binding energy of standard Co-MOF [33]. According to the previous report, the binding energy width equal to 16.2 eV between the main signals of the Co 2p_1/2_ and Co 2p_3/2_ doublet correspond to the Co^2+^ oxidation state [34].

**Figure 4:**
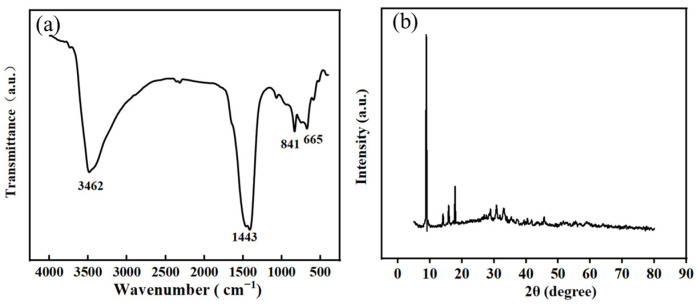
FTIR spectrum (**a**) and XRD pattern (**b**) of Co-MOF.

FTIR was used to further investigate the material’s functional group information. The aromatic ring structure and characteristic groups of Co-MOF, as demonstrated in Figure 4a, were well retained in the wavelength range from 400 cm^−1^ to 4000 cm^−1^. It is speculated that hydrogen bonds are formed between C=O and –OH and equalized the electron cloud density, thus causing the movement of the position of the C=O peak to lower wavelengths. Therefore, the significant stretching vibration peak of the C=O peak at 1443 cm^−1^ can be seen, as can the stretching vibration peak of the O-H functional group at 3462 cm^−1^ [35,36]. The crystal structures and phase identification of the materials were investigated by XRD analysis. Figure 4b presents the experimental XRD pattern for Co-MOF. As demonstrated, the diffraction peaks of Co-MOFs are consistent with the simulated Co-MOF (Cambridge Crystallographic Data Center, No. 153067) [37,38]. The diffraction peaks at 8.86°, 14.14°, 15.84° and 17.82° corresponded to Co-MOF [39], certifying that Co-MOF with well crystallization have been successfully synthesized.

### 2.2. Electrochemical Characterization of Co-MOF

**Figure 5:**
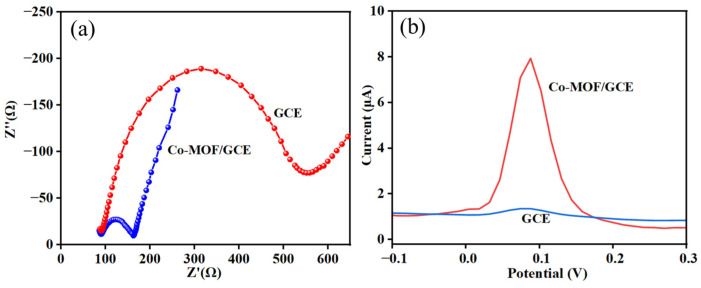
(**a**) EIS of bare GCE and Co-MOF/GCE in 0.1 M KCl containing 5 mM [Fe(CN)_6_]^3−^/^4−^, inner is the equivalent circuit; (**b**) Comparison of DPV responses of bare GCE and Co-MOF/GCE in 0.1 M PBS solution (pH 6.5) containing 10 μM DMY.

The electrochemical behaviors of the bare GCE and Co-MOF/GCE were measured by electrochemical impedance spectroscopy (EIS) in 0.1 M KCl containing 5 mM (Fe(CN)_6_) ^3−^/^4−^ solutions. As shown in Figure 5a, the resistance value of bare GCE is 520 Ω, while the resistance value of Co-MOF/GCE is 160 Ω. Due to the excellent conductivity of the Co metal, the organic skeleton material centered on metal Co significantly reduces the resistance value. Impedance difference between the bare GCE and the Co-MOF/GCE confirmed that Co-MOF material has been successfully modified on the surface of the GCE.

### 2.3. Electro-Oxidation Mechanism of DMY on Co-MOF/GCE

Differential pulse voltammetry (DPV) was used to analyze the property change of the electrode surface. In Figure 5b, the bare GCE showed an oxidation peak situated at a potential of 0.088 V with the current intensity of 0.510 μA in a 0.1 M PBS (pH 6.5) solution containing 10 μM DMY. However, when the bare GCE was modified with Co-MOF, the peak current significantly increased (7.185 μA), indicating that the synthesized Co-MOF can greatly amplify the electro-oxidation signal. This phenomenon is attributed to the fact that Co-MOF has multiple metal catalytic sites, which can interact with each other to produce a synergistic catalytic effect, and the metal element Co itself has a good electrical conductivity. Therefore, the Co-MOF/GCE is an ideal choice for the detection of DMY.

**Figure 6:**
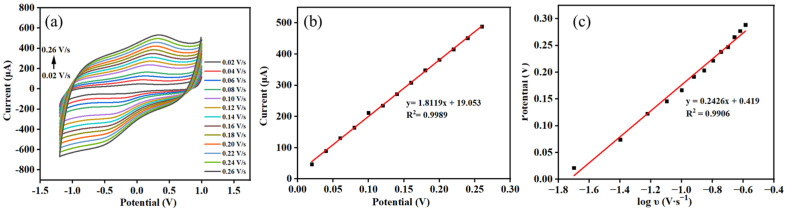
(**a**) CVs of the Co-MOF/GCE with scan rates ranging from 0.02 to 0.26 V·s^−1^ in 0.1 M PBS solution (pH 6.5) containing 10 μM DMY; (**b**) plot of peak current vs. the scan rate; (**c**) the linear relationship of *Ep* vs. *logυ*.

The cyclic voltammetry (CV) technique was used to examine the impact of the scan rates on the oxidation currents of DMY. Figure 6a shows the CVs of 10 μM DMY on the Co-MOF/GCE at different scan rates. The redox peak currents of DMY increased with the increase in scan rate in the range of 0.02−0.26 V·s^−1^. In addition, the reduction peak currents were linearly proportional to the scan rates, as seen in Figure 6b, where I_p_ = 1.8119υ + 19.053 (R^2^ = 0.9989), suggesting that the kinetics of the electrochemical oxidation–reduction reactions of DMY on the Co-MOF/GCE were an adsorption-controlled process [40]. Additionally, in Figure 6c, it can be seen that the relationship between the potentials and logs of the scan rates (υ) can be expressed by the equation: E_p_ = 0.2426 logυ + 0.419 (R^2^ = 0.9906), according to Laviron’s equation [41].

(1) $$E_{pa}=E^{0^{\prime }}+\frac{2.303RT}{(1-\alpha )nF}\log\upsilon$$

where the slope is equal to 2.303*RT*/(1−*α*)*nF*. As for a totally irreversible electrode process, the electron transfer coefficient (*α*) can be assumed as 0.5, and thus the electron transfer number (*n*) was calculated to be 1.15.

**Figure 7:**
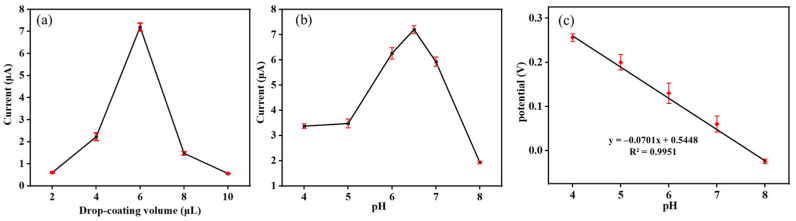
(**a**) Optimization of the content of Co-MOF; (**b**) optimization of pH; (**c**) the relationship between peak potential and pH.

It is commonly recognized that different parameters have a distinct influence on the outcomes of experiments. The modification amount of Co-MOF material has an important influence on the electrochemical response of DMY. By precisely controlling the drop coating amount, the electrochemical activity and electron transfer efficiency of the electrode can be significantly enhanced to ensure that the subsequent experiments can be carried out at a higher current level, which helps to enhance the sensitivity of the detection. In this work, 2, 4, 6, 8 and 10 μL of Co-MOF dispersion were drop-coated on the pretreated GCE, and the electrochemical signals of the Co-MOF/GCE were detected by using DPV for 10 μM DMY, and the optimal drop-coating amount of the material was obtained by comparison. As indicated in Figure 7a, the best Co-MOF drop-coating amount was 6 μL.

**Figure 8:**
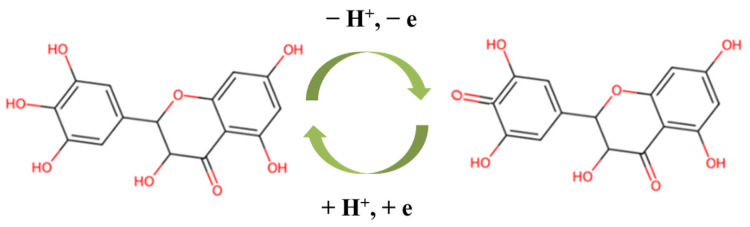
The plausible redox reaction of DMY.

The effect of pH value on the performance of the sensor is very important. The oxidation current values of 10 μM DMY on Co-MOF/GCE changed in 0.1 M PBS at different pH levels, as demonstrated in Figure 7b. From Figure 7b, we can see that the response current value increased for pH 4.0−6.5 and then decreased for pH 6.5−8. When the pH value was 6.5, the DMY oxidation peak current reached the maximum. Thus, pH 6.5 was chosen as the best pH condition. Furthermore, the oxidation peaks shift positively with the increase of the pH value at the range from pH 4.0 to 8.0. It can be seen from Figure 7c that the linear equation can be expressed as E_p_ = –0.0701pH + 0.5448 (R^2^ = 0.9951), which indicates that the oxidation reaction of DMY involves protons. The slope of the equation ΔE_p_/Δ_pH_ is –0.0701. According to equation [42], $E_{pa}=E^{0^{\prime }}-\frac{2.303RT}{nF}m\cdot pH$, *m* is the number of H^+^, and *m*/*n* is calculated as 1.20. The results show that the number of protons involved in the electrode reaction is the same as the number of electrons. Therefore, the number of electrons involved in the reduction reaction is 1.20. So, the proposed electro-oxidation mechanism of DMY on the Co-MOF/GCE surface may be expressed with the following (Figure 8).

### 2.4. Calibration Curve

**Figure 9:**
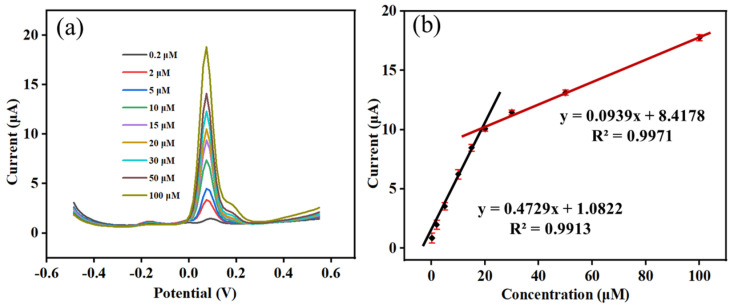
(**a**) DPVs of different concentrations of DMY on Co-MOF/GCE in 0.1 M PBS (pH 6.5); (**b**) calibration curve (*n* = 3).

Under the optimal detection conditions, the quantitative determination of DMY was performed in a 0.1 M PBS (pH 6.5) solution using DPV on Co-MOF/GCE. DMY solutions with concentrations ranging from 0.2 to 100 μM were prepared, and the relationship between the peak currents and the concentrations was analyzed. The results are shown in Figure 9. In the DPV response curves obtained after adding different concentrations of DMY to 0.1 M PBS, the peak current of the DPV response increased with the increase of DMY concentration. Two linear relationships were established in the range of 0.2−20 μM and 20−100 μM, as Ip (μA) = 0.4729*c* (μM) + 1.0822 (R^2^ = 0.9913) and Ip (μA) = 0.0939*c* (μM) + 8.4178 (R^2^ = 0.9971), respectively. The detection limit of DMY was 0.07 μM (signal-to-noise ratio = 3).

Table 1 summarizes the performance comparison of different electrochemical sensors for DMY determination in electrode-modified materials, linear ranges and detection limits. As listed in Table 1, the proposed sensor has a wide linear range in this work, implying that there is a wider application prospect. So, it is comparable to other methods documented in literature.

### 2.5. Selectivity, Repeatability and Stability of Co-MOF/GCE

To understand the repeatability of Co-MOF/GCE, it was placed in 0.1 M PBS containing 10 μM DMY and scanned by the DPV method. The response of the electrode to DMY was recorded, followed by removing the electrode and drying it naturally. The above steps were repeated and measured 5 times, and the relative standard deviation (RSD) of the response values was calculated. The results showed that the RSD of 5 measurements was 6.74% (Table 2)

The stability of Co-MOF/GCE was studied by comparing the performance changes of the same prepared modified electrode before and after 7 days. The Co-MOF was stored in a refrigerator at 4 °C for one week, and it was taken out and placed in 0.1 M PBS solution containing 10 μM DMY on the 7th day. The electrode response to DMY was recorded by scanning using DPV for 5 consecutive measurements, and the RSD of the measured values was calculated. The results are listed in Table 2. The initial response value of Co-MOF/GCE was 6.606 μA, and the response value was 6.286 μA after 7 days, which was 95.2% of the initial value with an RSD of 5.34%. These results indicated that the stability and repeatability of Co-MOF/GCE were good.

To evaluate the selectivity of Co-MOF for the detection of DMY, the interference test was done by adding mineral and metal ions and flavonoid analogs similar to dihydromyricetin to a 0.1 M PBS (pH 6.5) solution containing 10 μM DMY. The current changes of 10 μM DMY in the presence of different interfering substances were examined by adding several potentially interfering substances, including NaCl, KCl, MgSO_4_, CaSO_4_, glucose (Glu), quercetin (Qct) and genistein (Gnt). The current changes of DMY in the presence of different interfering substances were recorded, and each experiment had to be repeated more than five times to ensure the accuracy of the experiment.

**Figure 10:**
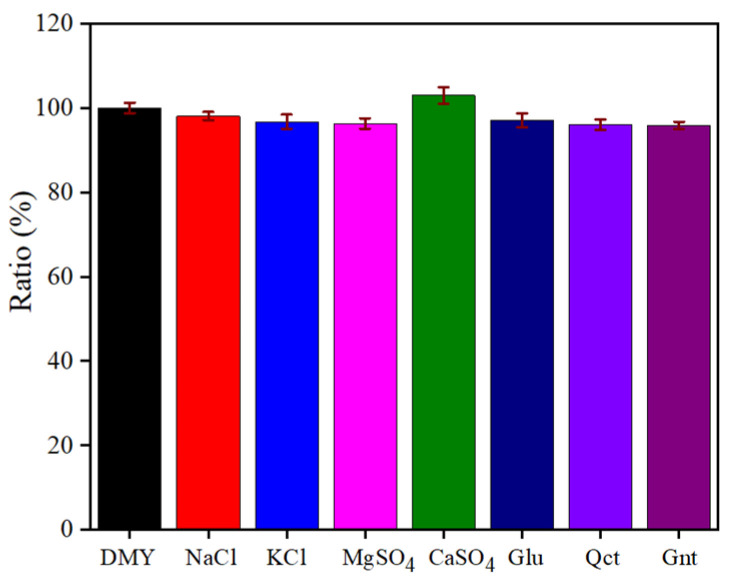
The influence of interfering substances on the peak currents of 10 μM DMY in 0.1 M PBS (pH 6.5).

As implied in Figure 10, the results show that the interference of NaCl, KCl, MgSO_4_, CaSO_4_, Glu, Que and Gnt on DMY (10 times in concentration) was within 5%, which were not significant and within the permissible range, indicating that the proposed Co-MOF/GCE demonstrated significant selectivity for DMY. Although myricetin is one of the main ingredients, the content of myricetin is 4–5 times lower than the content of DMY, and the peak potential is apart from the DMY. Therefore, the proposed Co-MOF/GCE is believed to be a good strategy for the selectivity of DMY.

### 2.6. Analysis of Ampelopsis grossedentata Sample

Using a freshly prepared electrode, DMY in the *Ampelopsis grossedentata* sample was detected to assess the proposed method’s practical applicability. The contents of DMY were determined by adding 10 μL of the pretreated *Ampelopsis grossedentata* sample solution in 0.1 M PBS (pH 6.5) solution, and then the detection was carried out by the DPV method, followed by the spiked recovery experiments. The results are listed in Table 3. Results show that the Co-MOF/GCE exhibited a strong electrochemical response to DMY. The same sample was also subjected to the HPLC technique. The standard’s test was used to compare the analytical findings from the HPLC method and the suggested method. The experiment was conducted with an Agilent 1260 Series liquid chromatography equipped with a UV–Vis detector set at 290 nm and a Hypersil C18 column (250 mm × 4.6 mm × 5.0 μm). The mobile phase consisted of methanol and 0.1% phosphate solution (28:72, *v*/*v*) flowing at a rate of 1.0 mL min^−1^, while the injection volume was 10 μL. The column temperature was 25 °C.

The results demonstrated that the Co-MOF/GCE electrochemical sensor showed significant utility and superior performance in the determination of DMY content, proving that the sensor could be applied to the determination of real samples.

## 3. Materials and Methods

### 3.1. Reagents

DMY, (>98.0%, AR), quercetin (>98.5%, AR) and genistein (>97.0%, AR) were provided by Aladdin Reagent Co., Ltd. (Shanghai, China). Cobalt nitrate hexahydrate (Co(NO_3_)_2_·6H_2_O), 1,4-benzenedi-carboxylic acid, N,N-dimethylformamide (DMF), ethanol (C_2_H_5_OH), potassium ferricyanide (K_4_(Fe(CN)_6_)), potassium ferricyanide (K_3_(Fe(CN)_6_)), potassium chloride (KCl), potassium dihydrogen phosphate (KH_2_PO_4_) and dipotassium hydrogen phosphate (K_2_HPO_4_) were obtained from Sinopharm Chemical Reagent Co., Ltd. (Shanghai, China). All reagents used in the experiment were of analytical grade. KH_2_PO_4_ and K_2_HPO_4_ diluted in deionized water were used to make phosphate buffer solution (PBS).

The *Ampelopsis grossedentata* sample was purchased from Youfenglai Selenium Eco-agriculture Technology Co., Ltd. (Enshi, China).

### 3.2. Instruments

A CHI-660E electrochemical workstation (Chenhua, Shanghai, China) was used to record the electrochemical measurements. The reference electrode was an Ag/AgCl (KCl saturated) electrode, while the counter electrode was a platinum electrode. The Co-MOF modified GCE (Co-MOF/GCE, Φ = 3 mm) was used as the working electrode. The source of all the electrodes was Chenhua Co., Ltd. Using an EDS (Oxford, UK), an FEI Inspect F50 SEM (Thermo Fisher, USA), G2 XPS (Thermo Fisher, USA), D8-A25 XRD (Bruker, Germany) and a VERTEX-70 FTIR (Bruker, Germany), the morphology and structure of the synthesized materials were characterized.

### 3.3. Synthesis of Co-MOF

The synthesis of Co-MOF was as per previous literature with a little modification [43]. First, 1.310 g Co(NO_3_)_2_·6H_2_O and 0.249 g 1,4-benzenedi-carboxylic acid were slowly added to 30 mL DMF and stirred at room temperature for 30 min. Then, the mixed solution was transferred into a 50 mL Teflon-lined autoclave and reacted at 150 °C for 12 h. After the reaction, the product was washed by DMF and ethanol, respectively. Finally, the centrifugally dried and pink powder Co-MOF was collected for the subsequent characterizations.

### 3.4. Preparation of Co-MOF/GCE Sensor

Before being used, the bare GCE was polished with alumina powder on chamois leather and then submerged in anhydrous ethanol and deionized water for 3 min, respectively. Using 0.5 M sulfuric acid solution, CV was used to activate the bare GCE. The dried electrode was coated with 6 μL of Co-MOF dispersion solution (1 mg mL^−1^), which was designated as Co-MOF/GCE.

### 3.5. Pretreatment of Actual Samples

Since the ethanol extract of DMY can improve the efficacy [44], the *Ampelopsis grossedentata* samples received pretreatment in compliance with the literature [45]. A total of 1 g of *Ampelopsis grossedentata* samples were combined with 10 mL ethanol (75%) for 10 min. Centrifugation was used to extract the *Ampelopsis grossedentata* supernatant. In order to conduct a spiking experiment, some *Ampelopsis grossedentata* samples and a certain volume of DMY standard concentration solution were mixed in 0.1 M PBS (pH 6.5).

### 3.6. Electrochemical Measurements

Each modified electrode was electrochemically characterized using 0.1 M KCl as the supporting electrolyte and 5 mM (Fe(CN)_6_)^3−/4−^ as the redox probe. The CV experiments were performed in the potential range from –0.2 to +1.0 V at the scanning rate of 100 mV s^−1^. The potential parameter of DPV experiment was from 0 to +1.4 V, the amplitude was 0.05 V, the pulse width was 0.06 s and the pulse period was 0.5 s. EIS was conducted at the open circuit voltage with the amplitude of 0.005 V and a frequency range from 1 Hz to 10^5^ Hz.

## 4. Conclusions

Based on the synthesis of Co-based MOF, a sensitive electrochemical sensor (Co-MOF/GCE) for DMY assessment was developed in this work. The Co-MOF/GCE has exceptional sensitivity and greatly boosts the electro-oxidation peak current of DMY. The prepared Co-MOF/GCE electrochemical sensor demonstrates a broad linear detection range of 0.20–100 μM with a low detection limit of 0.07 μM. The proposed sensor is also very stable and reproducible, making it suitable for application in the determination of DMY in *Ampelopsis grossedentata*.

## Figures and Tables

**Table 1:** Comparison between major characteristics of different sensors for DMY determination.

Modified Electrode	Linear Range (μM)	Detection Limit (μM)	Ref.
DNA/En/PGA ^1^/GCE	0.04–2	0.02	[22]
Nafion/SWNT ^2^/GCE	0.1–10	0.09	[23]
Nafion/graphene/GCE	0.08–20	0.02	[24]
Co-MOF/GCE	0.2–20, 20–100	0.07	This work

^1^ En/PGA: ethylenediamine/polyglutamic; ^2^ SWNT: single-walled carbon nanotube.

**Table 2:** Repeatability and stability of Co-MOF/GCE.

No.	Measured Initially (μA)	Average (μA)	RSD (%)	Measured after 7 days (μA)	Average (μA)	RSD (%)
1	6.083	6.606	7.18	5.732	6.286	5.34
2	7.005			6.325		
3	6.303			6.437		
4	7.196			6.307		
5	6.444			6.631		

**Table 3:** Determination of DMY in *Ampelopsis grossedentata* by Co-MOF/GCE and HPLC.

No.	Co-MOF/GCE				HPLC (μM)
	Detected (μM)	Added (μM)	Total (μM)	Recovery (%)	
1	3.005	5	8.155	103	3.213
2	3.545	5	9.072	111	3.447
3	3.277	5	8.840	111	3.283

## Data Availability

No new data were created or analyzed in this study.
